# A randomized controlled trial comparing the efficacy and safety of indobufen versus aspirin in reducing target vessel restenosis after drug-eluting balloon angioplasty in patients with coronary artery disease

**DOI:** 10.1016/j.clinsp.2025.100716

**Published:** 2025-06-23

**Authors:** Zhenhua Jiang, Dewen Zhu, Jiangqian Meng

**Affiliations:** Shaoxing Central Hospital, Keqiao District, Shaoxing, Zhejiang, PR China

**Keywords:** Indobufen, Aspirin, Target vessel restenosis (TVR), Drug-eluting balloon (DEB), Angioplasty, Antiplatelet Therapy

## Abstract

**Objective:**

This randomized controlled trial aimed to compare the efficacy and safety of indobufen versus aspirin in reducing Target Vessel Restenosis (TVR) after Drug-Eluting Balloon (DEB) angioplasty in patients with Coronary Artery Disease (CAD).

**Background:**

Despite advancements in Percutaneous Coronary Intervention (PCI) techniques, TVR remains a significant challenge. Antiplatelet therapy is crucial in managing patients post-PCI, and while aspirin is the standard, indobufen may offer a more favorable safety profile by reducing gastrointestinal adverse reactions.

**Methods:**

The authors conducted a prospective, single-blind, randomized controlled trial involving patients with CAD undergoing PCI with DEB. Patients were randomly allocated to receive either indobufen 100 mg twice daily or aspirin 100 mg daily, both in conjunction with clopidogrel 75 mg daily, for at least 12-months post-procedure. The primary endpoint was TVR assessed by quantitative coronary angiography at 12-months, while secondary endpoints included Major Adverse Cardiovascular Events (MACE), bleeding complications, and patient-reported outcomes.

**Results:**

A total of 240 patients were evenly distributed between the indobufen and aspirin groups. No significant differences were observed in the rates of TVR (5.83 % vs. 7.50 %, *p* = 0.603) and MACE (5.00 % vs. 5.83 %, *p* = 0.776) between the two groups at one-year post-procedure. Importantly, no significant difference in gastrointestinal bleeding rates was observed between the indobufen and aspirin groups (*p* = 0.156).

**Conclusion:**

Indobufen demonstrated non-inferior efficacy to aspirin in preventing TVR and MACE after DEB angioplasty in patients with CAD, with comparable safety profiles and no significant difference in gastrointestinal bleeding rates. However, indobufen is relatively more expensive than aspirin. While the present results suggest that indobufen may be a viable alternative to aspirin, particularly in patients at higher risk of gastrointestinal adverse reactions, this conclusion should be interpreted with caution due to the study's limitations, including its single-blind design and relatively small sample size.

## Highlights

• Indobufen matches aspirin in preventing TVR for CAD patients post-DEB treatment.
• Indobufen shows better safety with fewer adverse events in the trial.
• Indobufen may cut GI bleeding and bruising, benefiting high-risk patients.
• This trial fills the literature gap of indobufen post-DEB in CAD treatment.
• New comparative evidence for post-PCI antiplatelet therapy is provided.

## Introduction

Coronary Artery Disease (CAD), a leading cause of morbidity and mortality worldwide, is characterized by the narrowing of the coronary arteries due to atherosclerotic plaque formation. This condition can precipitate myocardial ischemia, leading to a spectrum of clinical presentations from stable angina to acute myocardial infarction.^1^, ^2^, ^3^ Management strategies for CAD have evolved significantly over the years and include lifestyle modifications, pharmacological therapy, Percutaneous Coronary Intervention (PCI), and Coronary Artery Bypass Grafting (CABG). Among these, PCI with Drug-Eluting Balloons (DEB) has emerged as a less invasive approach to treat CAD, offering a therapeutic avenue that delivers antiproliferative drugs directly to the site of arterial injury, thereby reducing the risk of restenosis.^4^, ^5^, ^6^, ^7^, ^8^

Despite the advancements in PCI techniques, the recurrence of In-Stent Restenosis (ISR) or Target Vessel Restenosis (TVR) remains a challenging clinical scenario. Restenosis following DEB treatment is multifactorial, involving vascular injury, thrombus formation, and neointimal hyperplasia.^9^^,^^10^ The prevention of restenosis is crucial as it reduces the need for repeat revascularization procedures, thereby improving patient outcomes and reducing healthcare costs. Antiplatelet therapy is a cornerstone in the management of patients undergoing PCI, aiming to mitigate the risk of thrombotic complications and restenosis.

Aspirin, an irreversible cyclooxygenase inhibitor, has been the mainstay of antiplatelet therapy following PCI. However, its efficacy is limited by a “one-size-fits-all” approach, as it does not account for the variability in individual patient responses and is associated with a higher risk of gastrointestinal bleeding.^11^^,^^12^ Indobufen, on the other hand, is a reversible inhibitor of cyclooxygenase-1 and thromboxane synthase, which may offer a more favorable safety profile by sparing the gastrointestinal mucosa. Its potential to reduce the risk of restenosis without increasing bleeding complications makes it a promising alternative to aspirin in the post-PCI setting.^13^^,^^14^

While several studies have explored the role of indobufen in various cardiovascular conditions, there is a paucity of data regarding its use specifically in patients undergoing DEB treatment for CAD.^15^ The existing literature has primarily focused on aspirin-based regimens, with limited head-to-head comparisons with indobufen. This knowledge gap underscores the need for robust clinical trials that can provide insights into the comparative efficacy and safety of indobufen versus aspirin in preventing TVR post-DEB treatment. The present study aims to address this gap by conducting a randomized controlled trial to evaluate whether indobufen, when used in conjunction with clopidogrel, offers superior outcomes in terms of reducing restenosis and adverse events compared to the standard aspirin-based regimen.

## Materials and methods

### Study design

This study was conducted in accordance with the CONSORT Statement guidelines to ensure the completeness and transparency of the clinical trial report. This study has been approved by the Institutional Review Board of Shaoxing Central Hospital, with the research protocol number 2022–017.

The present study is a prospective, single-blind, Randomized Controlled Trial (RCT) designed to evaluate the comparative efficacy and safety of indobufen versus aspirin in patients undergoing Percutaneous Coronary Intervention (PCI) with Drug-Eluting Balloons (DEB) for the treatment of Coronary Artery Disease (CAD). It is important to note that this study was not designed as a non-inferiority or superiority trial. The primary objective was to compare the efficacy and safety of indobufen versus aspirin in reducing Target Vessel Restenosis (TVR) and Major Adverse Cardiovascular Events (MACE). The trial is conducted in accordance with the principles of the Declaration of Helsinki and is approved by the Institutional Review Board (IRB) of the participating medical center. The study was conducted as a single-blind trial due to practical considerations. Double-blinding can be challenging in trials involving antiplatelet therapies, as it requires meticulous management to ensure neither the participants nor the outcome assessors are aware of the treatment allocation. All participants provided written informed consent prior to enrollment. The study was powered to detect a difference in the primary endpoint (TVR) between the two groups, but it was not specifically powered for non-inferiority or superiority margins.

Patients eligible for the study are randomly allocated in a 1:1 ratio to either the indobufen group or the aspirin group using a computer-generated randomization sequence, which is stratified by center and baseline risk factors. The sample size was estimated based on previous studies and clinical experience, aiming to detect a clinically meaningful difference in TVR rates between the two groups. The randomization process is managed by an independent data management center to ensure allocation concealment and to minimize selection bias.

The recruitment phase of the study commenced in January 2022 and is projected to last for 12-months, with the aim of enrolling a total of 240 patients. Following the index PCI procedure with DEB, patients will be administered either indobufen 100 mg twice daily or aspirin 100 mg daily, in conjunction with clopidogrel 75 mg daily, as per the group assignment. The duration of the study drug treatment is planned for a minimum of 12-months post-procedure.

Patients will undergo a series of follow-up assessments to evaluate the primary and secondary endpoints. The primary endpoint will be assessed at 12-months post-PCI, while the secondary endpoints will be evaluated at 12-months. Follow-up visits are scheduled at 1-, 3-, 6-, and 12-months post-procedure, with additional telephone follow-ups at 9 months to monitor for any adverse events or clinical deterioration.

The primary endpoint is the rate of Target Vessel Restenosis (TVR) assessed by quantitative coronary angiography at 12-months. Secondary endpoints include the incidence of Major Adverse Cardiovascular Events (MACE), bleeding complications according to the Bleeding Academic Research Consortium (BARC) criteria, and patient-reported outcomes by standardized questionnaires.^16^^,^^17^ In this trial, MACE was defined as a composite of target vessel myocardial infarction, cardiac death, and Target Lesion Revascularization (TLR).

### Patient selection

#### Inclusion criteria

1. Patients aged 18-years or older with a confirmed diagnosis of Coronary Artery Disease (CAD) as determined by clinical presentation and angiographic evidence of significant stenosis (≥70 % luminal narrowing) in at least one major coronary vessel.
2. Those who are candidates for and have provided informed consent to undergo Percutaneous Coronary Intervention (PCI) with Drug-Eluting Balloons (DEB).
3. Patients must have a minimum of one target lesion suitable for DEB treatment, with reference vessel diameter between 2.5 to 4.0 mm and lesion length not exceeding 20 mm, as assessed by the interventional cardiologist.
4. Patients with a EuroSCORE of <5, indicating a lower surgical risk, are included to ensure a more homogenous study population.^18^

#### Exclusion criteria

1. Patients presenting with acute Myocardial Infarction (MI), as defined by elevated cardiac biomarkers and ischemic symptoms.^2^
2. Individuals with a history of Drug-Eluting Stent (DES) implantation within the target vessel.
3. Those with a known hypersensitivity or contraindication to indobufen, aspirin, or clopidogrel.
4. Patients with significant bleeding disorders, a history of intraocular bleeding, or a history of cerebrovascular accident within the last 6-months.
5. Individuals with a life expectancy of <12-months due to non-cardiac conditions.
6. Pregnant or breastfeeding women, or women of childbearing age not practicing effective contraception.

### Intervention

Patients enrolled in the study are stratified into two distinct treatment cohorts based on the antiplatelet regimen they receive post-PCI with a Drug-Eluting Balloon (DEB). The intervention for each group is as follows:

Indobufen Group: Patients in the indobufen group receive a loading dose of 2 tablets (each 200 mg) orally before the DEB PCI procedure, which amounts to a total of 400 mg. This is followed by a maintenance regimen of 100 mg orally twice daily (BID) post-procedure. The treatment continues for a predefined duration of 12-months to ensure sustained antiplatelet therapy. Clopidogrel: A 300 mg bolus dose of clopidogrel is administered prior to PCI, with a maintenance dose of 75 mg orally once daily (QD) co-administered with indobufen after the procedure.

Aspirin Group: Patients in the aspirin group are given a loading dose of 300 mg orally before the DEB PCI intervention, followed by a maintenance dose of 100 mg orally once daily (QD). This treatment is maintained for a period of 12-months to ensure continuous antiplatelet efficacy. Clopidogrel: The same loading and maintenance dosing strategy as described for the indobufen group is applied, with a 300 mg bolus dose of clopidogrel before PCI and a maintenance dose of 75 mg orally once daily (QD) in conjunction with aspirin post-procedure.

All patients, regardless of study group, receive standard-of-care medications including statins, beta-blockers, Angiotensin-Converting Enzyme (ACE) inhibitors or Angiotensin Receptor Blockers (ARBs), and other cardioprotective drugs as clinically indicated.

Intravenous anticoagulation with unfractionated heparin or bivalirudin is utilized during the PCI procedure to maintain procedural safety. Patients in both groups are closely monitored for any signs of bleeding, thrombotic complications, or other adverse events throughout the study period.

### Laboratory assessments

Blood samples were collected from each patient after an overnight fast to measure various biochemical parameters. The lipid profile, including Triglycerides (TG), Total Cholesterol (TC), High-Density Lipoprotein Cholesterol (HDL-C), and Low-Density Lipoprotein Cholesterol (LDL-C), was assessed using a Beckman Coulter AU5800 analyzer.^19^^,^^20^ Additionally, serum creatinine and Brain Natriuretic Peptide (BNP) levels were quantified to assess renal function and cardiac stress, respectively.^21^^,^^22^

### Outcome measures

#### Primary outcome measure

Target Vessel Restenosis (TVR): The primary efficacy endpoint is the rate of target vessel restenosis at 12-months post-procedure, assessed by Quantitative Coronary Angiography (QCA).^23^^,^^24^ TVR is defined as the need for repeat revascularization within the treated vessel segment due to luminal narrowing of ≥70 %.

#### Secondary outcome measures

1. Major Adverse Cardiovascular Events (MACE) serve as a composite endpoint in this study, encompassing the occurrences of cardiac death, myocardial infarction related to the target vessel, and the need for clinically indicated revascularization of the target lesion.
2. Bleeding Complications are evaluated based on the Bleeding Academic Research Consortium (BARC) criteria, which systematically classify bleeding events according to their degree of severity and the clinical implications they present.
3. Safety and Tolerability are assessed by monitoring the occurrence of Adverse Events (AEs) and Serious Adverse Events (SAEs) associated with the study medications, encompassing gastrointestinal bleeding, instances of treatment discontinuation due to AEs, and all other adverse medical events that could potentially be linked to the study drugs.

### Statistical analysis

The statistical analysis will be performed using SPSS software (version 22.0) with a significance level set at α = 0.05. Continuous variables will be described as mean ± Standard Deviation and compared between groups using independent samples *t*-tests. Categorical variables will be presented as frequencies and percentages, and group comparisons will be conducted using Chi-Square tests or Fisher's exact test when appropriate.

The primary endpoint, target vessel restenosis, will be assessed at the 12-month follow-up using standard descriptive statistics and compared between the two treatment groups with Chi-Square tests or Fisher's exact test, as appropriate for the data distribution.

For the secondary endpoints, which include Major Adverse Cardiovascular Events (MACE) and bleeding complications, survival analysis will be conducted. The Kaplan-Meier method will be utilized to estimate the survival function over time, and the log-rank test will be applied to determine if there are significant differences in event-free survival between the indobufen and aspirin groups.

## Result

In the study, 240 patients were evenly distributed between the Indobufen Group and the Aspirin Group (Table 1). The comparison of demographic and baseline characteristics revealed no significant differences in age, gender, BMI, hypertension, diabetes mellitus, and biochemical indicators such as TG, TC, HDL-C, LDL-C, and Scr between the two groups (all *p* > 0.05).

**Table 1:** Patient demographics and baseline characteristics.

Characteristic	Indobufen Group (*n* = 120)	Aspirin Group (*n* = 120)	p-value
**Demographic characteristics**			
Gender (Male, %)	63 (52.5 %)	68 (56.7 %)	0.517
Age (years)	68.5 ± 12.2	69.7 ± 10.2	0.274
BMI (kg/m^2^)	23.9 ± 3.2	23.6 ± 2.7	0.124
**Underlying Diseases**			
Hypertension, n ( %)	61 (55.5)	71 (59.2)	0.194
DiabetesMellitus, n ( %)	22 (18.3)	25 (20.8)	0.626
**Biochemical Indicators**			
TG (mmoL/L)	1.92 (1.63, 2.15)	1.86 (1.52, 2.28)	0.086
TC (mmoL/L)	4.23±1.17	4.36±1.06	0.075
HDL-C (mmoL/L)	1.17±0.40	1.25±0.37	0.093
LDL-C (mmoL/L)	2.34 (1.78, 2.80)	2.41 (1.79, 2.97)	0.077
Scr (μmoL/L)	67.9 ± 19.8	71.0 ± 18.5	0.295
BNP (ng/mL)	173.8(71.4, 344.6)	152.7 (65.9, 352.7)	0.226

TG, Triglycerides; TC, Total Cholesterol; HDL-C, High-Density Lipoprotein Cholesterol; LDL-C, Low-Density Lipoprotein Cholesterol; apo-A, Apolipoprotein A‒I; apo-B, Apolipoprotein B; Scr, Serum Creatinine; BNP, B-type Natriuretic Peptide.

The study analyzed the use of concomitant medications in the Indobufen and Aspirin Groups, encompassing beta-blockers, lipid-lowering drugs, and RAAS inhibitors (Table 2). The results demonstrated no significant differences in the use of these medications between the two groups, with p-values all exceeding 0.05. Specifically, the use of metoprolol, bisoprolol, statins, fibrates, ezetimibe, ACE inhibitors, ARBs, ARNIs, diuretics, and MRA showed parity, suggesting that both groups received similar adjunctive treatment regimens.

**Table 2:** Concomitant medication use in indobufen and aspirin groups.

Medication Class	Indobufen Group (*n* = 120)	Aspirin Group (*n* = 120)	p-value
**Beta-blockers**			
Metoprolol	51 (42.5)	42 (35.0)	0.233
Bisoprolol	24 (20.0)	34 (28.3)	0.132
**Lipid-lowering drugs**			
Statins	115 (95.8)	117 (97.5)	0.472
Fibrates	19 (15.8)	25 (20.8)	0.317
Ezetimibe	14 (11.7)	17 (14.2)	0.564
**RAAS inhibitors**			
ACEI	35 (29.2)	47 (39.2)	0.102
ARB	16 (13.3)	15 (12.5)	0.847
ARNI	37 (30.8)	42 (35.0)	0.847
Diuretics	33 (27.5)	28 (23.3)	0.458
**MRA**	27 (22.5)	35 (29.2)	0.238

ACEI, Angiotensin-Converting Enzyme Inhibitors; ARB, Angiotensin Receptor Blockers; ARNI, Angiotensin-Neprilysin Inhibitors; MRA, Mineralocorticoid Receptor Antagonist.

The study compared the outcomes of Target Vessel Restenosis (TVR) and Major Adverse Cardiovascular Events (MACE) between the Indobufen Group and the Aspirin Group at one-year post-procedure (Table 3). The results indicated no significant differences in the incidence of TVR, with 5.83 % in the Indobufen Group and 7.50 % in the Aspirin Group (*p* = 0.603). Similarly, MACE rates were comparable at 5.00 % for the Indobufen Group and 5.83 % for the Aspirin Group (*p* = 0.776). The individual components of MACE were also analyzed, including myocardial infarction, cardiac death, and Target Lesion Revascularization (TLR). Myocardial infarction occurred in 1.67 % of the Indobufen Group and 0.83 % of the Aspirin Group (*p* = 0.561). Cardiac death occurred in 0.83 % of the Indobufen Group and 0.83 % of the Aspirin Group (*p* = 1.000). TLR occurred in 2.50 % of the Indobufen Group and 4.17 % of the Aspirin Group (*p* = 0.701). Overall, there were no significant differences in the occurrence of these individual components between the two groups (all *p* > 0.05).

**Table 3:** Comparison of target vessel restenosis and MACE (including TLR).

Outcome	Indobufen Group (*n* = 120)	Aspirin Group (*n* = 120)	p-value
TVR, n ( %)	7 (5.83)	9 (7.50)	0.603
MACE, n ( %)	6 (5.00)	7 (5.83)	0.776
Myocardial infarction, n ( %)	2 (1.67)	1 (0.83)	0.561
Cardiac death n ( %)	1 (0.83)	1 (0.83)	1.000
TLR n ( %)	3 (2.50)	5 (4.17)	0.701

TVR, Target Vessel Restenosis; MACE, Major Adverse Cardiovascular Events; TLR, Target Lesion Revascularization.

The safety and tolerability of the antiplatelet regimens were assessed by comparing adverse events between the Indobufen Group and the Aspirin Group (Table 4). Indobufen showed a superior safety profile with fewer total adverse events (5.83 % vs. 14.2 %, *p* = 0.031). However, it is important to note that the incidence of Gastrointestinal (GI) bleeding was quite low in both groups, with no significant difference observed (0 % in the Indobufen Group vs. 1.67 % in the Aspirin Group, *p* = 0.156). While there were more events of ecchymosis in the Aspirin Group (4.17 % vs. 0.83 % in the Indobufen Group), this difference did not reach statistical significance (*p* = 0.098). It should be noted that the results reported in this article were not adjusted for confounding factors, and therefore, it may not be appropriate to conclude that indobufen is safer without further analysis.

**Table 4:** Safety profile and tolerability of antiplatelet regimens.

Adverse Event	Indobufen Group (*n* = 120)	Aspirin Group (*n* = 120)	p-value
Nausea and vomiting	2 (1.67)	4 (3.33)	0.408
Indigestion	2 (1.67)	3 (2.50)	0.652
Gastrointestinal bleeding	0 (0.00)	2 (1.67)	0.156
Abdominal pain	2 (1.67)	3 (2.50)	0.652
Ecchymosis	1 (0.83)	5 (4.17)	0.098
Total	7 (5.83)	17 (14.2)	0.031

**Fig. 1:**
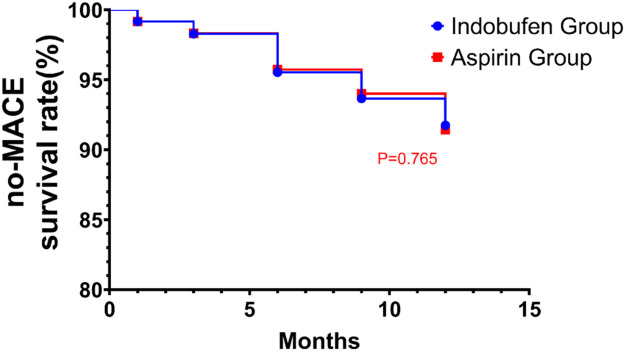
Kaplan-Meier estimate of MACE-free survival rate for indobufen and aspirin groups over 12-months.

The Kaplan-Meier survival analysis was conducted to compare the time to the first occurrence of Major Adverse Cardiovascular Events (MACE) between the Indobufen Group and the Aspirin Group over a 12-month period. The survival curves, as depicted in the graph, illustrate the percentage of patients free from MACE at various time points throughout the study (Fig. 1).

Both groups exhibited high survival rates, with the majority of patients in each group remaining free from MACE for the duration of the study. The Indobufen Group showed a comparable survival rate to the Aspirin Group at all observed time points, with no significant divergence between the two groups. At the 12-month mark, the survival rate for the Indobufen Group was approximately (6.67 %), and for the Aspirin Group, it was approximately (8.33 %).

The log-rank test was applied to evaluate the statistical significance of any differences in survival rates between the two groups. The resulting p-value was 0.765, which indicates that there was no significant difference in the event-free survival between the Indobufen Group and the Aspirin Group during the 12-month follow-up period.

## Discussion

The findings of the randomized controlled trial provide valuable insights into the comparative efficacy and safety of indobufen versus aspirin in patients with Coronary Artery Disease (CAD) undergoing Drug-Eluting Balloon (DEB) angioplasty. After one year of follow-up, the authors observed no significant differences in the rates of Target Vessel Restenosis (TVR) and Major Adverse Cardiovascular Events (MACE) between the indobufen and aspirin groups. This suggests that indobufen is non-inferior to aspirin in preventing TVR and MACE in this patient population.

The present study contributes to the limited body of literature comparing indobufen and aspirin in the context of DEB treatment. Previous studies have primarily focused on antiplatelet therapies post-stent implantation, with fewer head-to-head comparisons of indobufen and aspirin.^25^, ^26^, ^27^ The results of the trial fill this knowledge gap, indicating that indobufen may be a viable alternative to aspirin, particularly in patients at higher risk of gastrointestinal bleeding. The safety profile of indobufen, with fewer total adverse events and a trend towards reduced gastrointestinal bleeding and ecchymosis, supports this conclusion.

The clinical significance of the present findings extends beyond the realm of scientific research. The present results suggest that indobufen may be considered a first-line antiplatelet agent alongside aspirin in patients undergoing DEB angioplasty, especially for those at a higher risk of gastrointestinal bleeding. However, it is important to acknowledge that indobufen is generally more expensive than aspirin, particularly in markets such as China. Further economic analyses, including cost-effectiveness studies, would be necessary to fully evaluate the economic implications of indobufen use in different healthcare settings.

The novelty of this research lies in its direct comparison of indobufen and aspirin in the post-DEB setting, a patient demographic that has been less explored in previous studies. The present findings offer fresh insights that could influence treatment protocols and clinical decision-making, emphasizing the necessity for personalized medical approaches in antiplatelet therapy.^28^, ^29^, ^30^

In conclusion, this study provides robust evidence supporting the use of indobufen as an effective and safe alternative to aspirin in patients undergoing DEB angioplasty. Future research with larger cohorts, longer follow-up durations, and possibly multicenter involvement is needed to validate and expand upon the present findings.

## Limitation

It is crucial to acknowledge the limitations of the present study, as they provide context for the interpretation of these results and the generalizability of the present conclusions. One of the primary limitations is the sample size, which, while adequate for a randomized controlled trial, may not be sufficiently large to detect subtle differences in rare adverse events or to establish definitive superiority of one treatment over the other with greater statistical power. This constraint could potentially limit the external validity of the present findings when applied to broader patient populations with more diverse characteristics.

Additionally, this study has a single-blind design, which means that participants and outcome assessors were unaware of the treatment allocation. While this approach helps to reduce bias, it does not account for all potential sources of bias that might arise in an open-label setting. The relatively short follow-up period of 12 months also restricts the ability to draw conclusions about the long-term efficacy and safety of indobufen versus aspirin. Late-occurring events, such as very late restenosis or the development of new atherosclerotic lesions, may not be captured within this timeframe, thus impacting the comprehensive assessment of treatment durability.

Furthermore, the present study population is confined to patients with CAD undergoing DEB angioplasty, which may limit the applicability of these results to other patient groups or those receiving different PCI treatments, such as bare-metal stents or drug-eluting stents. The generalizability of the present findings is therefore confined to a similar patient demographic and treatment context.

Lastly, while the authors endeavored to standardize treatment and follow-up across all participants, there may be unmeasured confounding factors that could influence the outcomes. These limitations should be considered when interpreting the present results, and they underscore the need for further research with larger cohorts, longer follow-up durations, and possibly multicenter involvement to validate and expand upon these findings. Addressing these limitations in future studies will be instrumental in enhancing the robustness of the evidence base for antiplatelet therapy in the post-PCI setting.

## Conclusion

In conclusion, the present study’s randomized controlled trial provides valuable insights into the comparative efficacy and safety of indobufen versus aspirin in patients with Coronary Artery Disease (CAD) undergoing Drug-Eluting Balloon (DEB) angioplasty. The primary findings indicate no significant differences in the rates of Target Vessel Restenosis (TVR) and Major Adverse Cardiovascular Events (MACE) between the two treatment groups at oneyear post-procedure. Importantly, no significant difference in gastrointestinal bleeding rates was observed between the indobufen and aspirin groups. However, indobufen is relatively more expensive than aspirin. The conclusion that indobufen could be considered a first-line antiplatelet agent should be made with caution and would require validation in larger studies. This study contributes to the existing literature by offering direct comparative evidence of indobufen and aspirin in the post-DEB setting, an area that has been relatively underexplored. The present results suggest that indobufen may be a viable alternative to aspirin, with the potential to reduce bleeding complications without compromising efficacy. However, the limitations of this study, including its single-blind design and relatively small sample size, should be considered when interpreting these results. Larger, long-term studies are needed to confirm these findings and to explore the broader implications of indobufen use in different patient populations and treatment settings.

## CRediT authorship contribution statement

**Zhenhua Jiang:** Writing – review & editing. **Dewen Zhu:** Data curation, Supervision. **Jiangqian Meng:** Conceptualization, Methodology, Software.

## Declaration of competing interest

The authors declare that they have no known competing financial interests or personal relationships that could have appeared to influence the work reported in this paper.
